# Measured versus calculated LDL-cholesterol in subjects with type 2 diabetes

**DOI:** 10.12669/pjms.324.9896

**Published:** 2016

**Authors:** Asher Fawwad, Rubina Sabir, Musarrat Riaz, Hassan Moin, Abdul Basit

**Affiliations:** 1 Asher Fawwad, PhD. Associate Professor, Senior Research Scientist, Research Department, Baqai Institute of Diabetology and Endocrinology, Baqai Medical University, Karachi Pakistan; 2 Rubina Sabir, M.Sc. Laboratory Manager, Clinical and Research Laboratory, Baqai Institute of Diabetology and Endocrinology, Baqai Medical University, Karachi Pakistan; 3 Musarrat Riaz, F.C.P.S. Assistant Professor, Consultant Physician, Department of Medicine, Baqai Institute of Diabetology and Endocrinology, Baqai Medical University, Karachi Pakistan; 4 Hassan Moin, M.Sc. Statistician, Research Department, Baqai Institute of Diabetology and Endocrinology, Baqai Medical University, Karachi Pakistan; 5 Abdul Basit, F.R.C.P. Professor of Medicine, Department of Medicine, Baqai Institute of Diabetology and Endocrinology, Baqai Medical University, Karachi Pakistan

**Keywords:** Type 2 Diabetes, LDL

## Abstract

**Objective:**

There is a strong positive association between increased low-density lipoprotein cholesterol (LDL-C) and coronary heart disease (CHD). The accuracy of LDL-C estimation is essential and critically important. The aim of present study was to compare calculated LDL-C with direct homogeneous assay in patients with type 2 diabetes.

**Methods:**

This observational study was carried out at Baqai Institute of Diabetology and Endocrinology (BIDE) from January 2011 to December 2013. A total of 9620 patients with type 2 diabetes were included in the study. Fasting blood glucose, total Cholesterol, triglyceride, HDL cholesterol and LDL cholesterol were obtained using standard methods. Calculated LDL-C was obtained by Friedewald formula.

**Results:**

Mean difference of measured and calculated LDL-C was found to be -0.25, 6.63 and 46.55 mg/dl at triglyceride levels < 150 mg/dl, 150 - 400 mg/dl and ≥ 400 mg/dl, respectively. The result shows that the difference between measured and calculated LDL-C increases as the triglyceride level increases.

**Conclusions:**

The findings of our study suggested that calculated LDL-C was lower, as compared to measured LDL-C, which may cause misclassifications that may have an impact on therapeutic decisions in patients with diabetes. Calculated LDL-C may depend on triglyceride levels so LDL-C should be measured by direct assay in routine clinical laboratories.

## INTRODUCTION

Coronary Artery Disease is the leading cause of death worldwide. The level of low-density lipoprotein cholesterol (LDL-C) is one of the primary key predictor of atherosclerosis and coronary heart disease (CHD) risk.^1^-^3^ There is a strong positive association between increased LDL-C and CHD.^3^ LDL- cholesterol is used in clinical decision making guidelines to reducing cardiovascular risk events.^1^ According to National Cholesterol Education Programme’s (NCEP) Adult Treatment Panel III (ATP III) recommendations LDL-C is a primary risk factor for cardiovascular disease (CVD).^2^ About 1% reduction in LDL can reduce the risk of CAD by 1%.^4^ Pakistani population is also at high risk of increasing incidence of Coronary Artery Disease (CAD).^2^

Patients with diabetes mellitus are considered as a high risk for cardiovascular disease.^5^ Abnormal lipid profiles and increased levels of low density lipoproteins (LDL) are two components of the atherogenic profile seen in diabetes mellitus.^6^,^7^

In the view of all above findings the accuracy of LDL-C estimation is essential and critically important.^3^ Inaccurate estimation LDL-C can cause misclassification of patients into an inappropriate risk category.^1^

As measurement of LDL-C by direct method is expensive, so it is estimated by Friedewald equation in clinical and research settings worldwide.^1^,^2^ Friedewald equation have some limitations including requirement for fasting, analytical variability and invalidity in samples with triglyceride (TG) > 400 mg/dl and certain type of hyperlipidemias. Some studies have shown that the accuracy of this formula declines as triglyceride increases beyond 177 mg/dl.^1^,^2^ Inaccurate results of LDL-C may obtain by Friedewald equation, in conditions like type 2 diabetes mellitus.^1^

The reference method for measurement of LDL-C concentration combines with ultra centrifugation-polianion precipitation/Beta Quantification (ßQ).^2^-^4^ A new but somewhat expensive measured homogenous assays for LDL-C determination in serum also certified by NCEP and Cholesterol Reference Method Laboratory Network of Centre for Disease Control and Prevention for use in routine clinical laboratories is also available.^2^,^3^

The aim of present study was to compare calculated LDL-C with measured homogeneous assay in patients with type 2 diabetes.

## METHODS

This observational study was conducted in patients with type 2 diabetes from January, 2011 to December, 2013 at the outpatient department (OPD) and indoor ward of Baqai Institute of Diabetology and Endocrinology (BIDE), a tertiary care diabetes centre in Karachi Pakistan.

This is a retrospective study based on the hospital data records..

### Anthropometric measurements

Height and weight were measured to calculate Body mass index (BMI) as a ratio of weight (kg) to height squared (m^2^). BMI was categorized as normal; between 18.0-22.9 kg/m^2^; overweight between 23.0-24.9 kg/m^2^ and obese ≥ 25.0 kg/m^2^.^8^ Blood pressure of the participants was monitored by mercury sphygmomanometer in a sitting position using standard method.^9^

### Hypertension

Hypertension was defined as blood pressure >130/85 mmHg. This category includes patients taking antihypertensive medicines, even if treatment achieves a blood pressure level that is within target range.

### Biochemical tests

Biochemical analyses were carried out at clinical and research laboratory of BIDE. Blood was collected by venepuncture from all subjects using sterilized disposable vaccutainer needles in gel (for lipids), Sodium fluoride (for glucose) and EDTA K2 (for HbA1c) containing vaccutainer tubes.

### Blood glucose (GOD PAP method)

Fasting blood glucose was determined after enzymatic oxidation in the presence of glucose oxidase. The hydrogen peroxide formed reacts, under catalysis of peroxidase with phenol and 4-aminophenazone to form a red violet quinoneimine dye as indicator.^1^,^10^

### Total cholesterol (CHOD-PAP method)

Serum total cholesterol in Cholesterol was determined after enzymatic hydrolysis and oxidation, indicator quinoneimine is formed from hydrogen peroxide and 4-aminoantipyrine in the presence of phenol and peroxidase.^2^,^10^

### Triglyceride (GPO-PAP method)

Triglycerides were determined after enzymatic hydrolysis with lipases. The indicator was a quinoneimine formed from hydrogen peroxide, 4-aminop- henazone and 4-cholorophenol under the under the catalytic influence of peroxidase.^2^,^10^

### HDL–cholesterol (Homogeneous enzymatic colorimetric method)

HDL–cholesterol Immuno FS, a homogenous method for HDL–cholesterol measurement. Antibodies against human lipoproteins were used to form antigen – antibody complexes with LDL (low density lipoproteins), VLDL (very low density lipoproteins) and Chylomicrons in a way that only HDL–cholesterol is selectively determined by an enzymatic cholesterol measurement.^2^,^10^

### LDL–cholesterol (Direct method)

There are two steps involve in the estimation of LDL cholesterol by direct method. In first step, when a sample mixed with reagent 1 (MES buffer, ph 6.3, detergent 1, cholesterol esterase, cholesterol oxidase, peroxidase, 4-Amino-Antipyrine and ascorbic acid), non LDL lipoproteins are solubilized by detergent one and release cholesterol is subject to enzymatic reaction to be eliminated. In 2^nd^ step reagent 2 (MES buffer, ph 6.3, detergent 2, N,N-bis-m-tolidine-disodium) is added, LDL is solubilized by detergent 2, then LDL cholesterol is measured by enzymatic reactions.

### HbA1c (D-10)

Blood sample of 2ml volume is drawn in vaccutainer containing EDTA. Prime and calibrate D-10 with primer and calibrator provided with kit. The D-10 automatically calibrates itself with calibrator loaded. Place sample in rack and put the rack in D-10. Analyzer is then started to obtain results.

### LDL-cholesterol (formula)

LDL-C can be estimated by Friedewald equation

[LDL-C] = [TC] - [HDL-C] - [TG/5]

Here, LDL-C = Low density lipoprotein

HDL-C = High density lipoprotein

TC = Total cholesterol

and, TG = Triglyceride

The approximate value of LDL-C obtained is in mg/dl. The above equation can be modified by dividing TG with 2.2 in order to obtain LDL-C in mmol/l.

### Statistical analysis

Statistical analysis was performed on Statistical Package for Social Sciences (SPSS), version 17.0. All the continuous data was presented as Mean ± SD and categorical data as frequency (percentage). Independent t-test or chi-square test were used for finding difference between male and female group for anthropometric, clinical and lipid variables, whereas paired t-test was used to find mean difference between measured and calculated LDL-C. P<0.05 was considered statistically significant.

## RESULTS

Anthropometric characteristics of study population are shown in Table-I. A total of 9620 subjects (5425 males and 4195 females) were included in the study. Mean ages of study subjects was 50.54 ± 11.86, male 51.06 ± 11.89 and female 49.86 ± 11.78 years. BMI was found to be statistically significant (p<0.05). Systolic blood pressure was significantly higher in male diabetics as compare to female diabetic subjects (p<0.05). The mean of total cholesterol and HDL cholesterol was found to be statistically significant (p<0.05). All the variables had statistically significant difference except for triglyceride and HbA1c.

**Table-I:** Characteristics of anthropometric, clinical and lipid variables of subjects.

Variables	Male	Female	Overall
n	5425	4195	9620
Age (years)*	51.06 ± 11.89	49.86 ± 11.78	50.54 ± 11.86
Body mass index (kg/m^2^)*	26.90 ± 5.06	29.12 ± 6.23	27.87 ± 5.71
Obesity*			
Non-obese	1767 (37.3%)	900 (24.6%)	2667 (31.8%)
Obese	2972 (62.7%)	2754 (75.4%)	5726 (68.2%)
Systolic blood pressure (mmHg)*	126.86 ± 22.24	130.90 ± 25.23	128.60 ± 23.66
Diastolic blood pressure (mmHg)	80.46 ± 12.55	80.21 ± 13.62	80.36 ± 13.02
Hypertension*			
Yes	2066 (40.4%)	1876 (48.5%)	3942 (43.9%)
No	3053 (59.6%)	1990 (51.5%)	5043 (56.1%)
Fasting blood glucose (mg/dl)*	170.25 ± 65.06	187.76 ± 87.35	177.71 ± 75.79
HbA1c (%)	9.52 ± 2.31	9.47 ± 2.34	9.50 ± 2.32
Cholesterol (mg/dl)*	163.21 ± 47.46	174.46 ± 49.49	168.09 ± 48.67
Triglyceride (mg/dl)	159.76 ± 129.81	160.35 ± 112.99	160.02 ± 122.78
HDL-C (mg/dl)*	33.34 ± 8.99	36.81 ± 9.55	34.85 ± 9.40
LDL-C measured (mg/dl)*	101.37 ± 35.59	108.96 ± 37.89	104.68 ± 36.8
Dyslipidemia*			
Yes	2692 (49.6%)	2396 (57.1%)	5088 (52.9%)
No	2733 (50.4%)	1799 (42.9%)	4532 (47.1%)

Data presented as Mean ± S.D or n (%)

* Denotes p-value < 0.05

P-value <0.05 was considered statistically significant.

**Fig.1:**
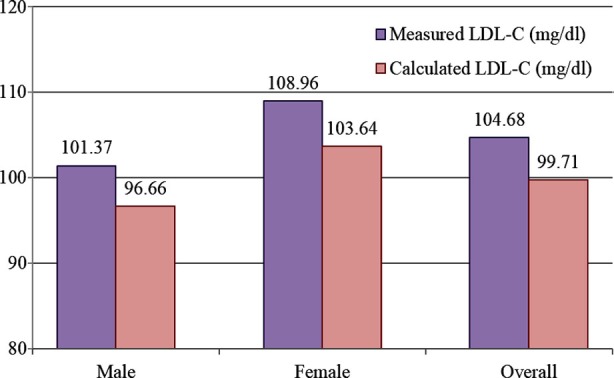
Comparison of measured and calculated LDL-C (by formula) P-value < 0.05 was considered statistically significant. P-value was found to be statistically significant in all cases.

Fig.1 shows the comparison of measured and calculated LDL-C in male and female subjects. It was observed that the measured LDL-C of the female diabetic patients was statistically significant as compare to male diabetic patients (p<0.05). The situation remains same in case of overall analysis.

**Fig.2:**
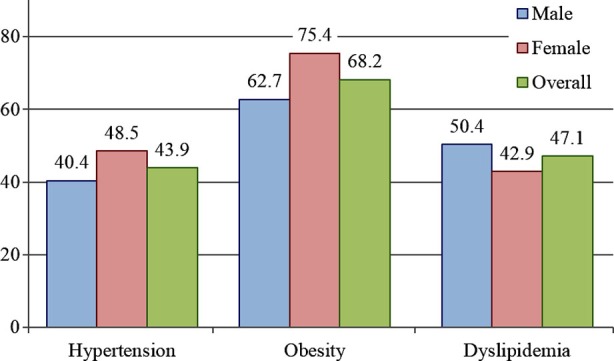
Frequency of dyslipidemia, obesity and hypertensive subjects. P-value < 0.05 was considered statistically significant P-value was found to be statistically significant in all cases.

Comparison of measured LDL-C and calculated LDL-C at different triglyceride levels are shown in Table-II. At low triglycerides levels, measured LDL-C and calculated LDL-C levels were 97.40 ± 32.87 and 97.65 ± 36.16 mg/dl respectively, having no statistically significant difference. But when triglycerides level ranges from 150 to 400 mg/dl, a significant increase in mean difference between measured LDL-C (115.71 ± 35.62 mg/dl) and calculated LDL-C level (109.08 ± 41.64 mg/dl) was found to be 6.63 ± 26.43 mg/dl. This difference then increases to 46.55 ± 55.36 mg/dl as triglyceride level is beyond 400 mg/dl (p<0.0001).

**Table-II:** Comparison of measured and calculated LDL-C (by formula).

	Triglyceride (mg/dl)		

	< 150 (mg/dl)	150-400 (mg/dl)	≥ 400 (mg/dl)
LDL-C (Measured)	97.40 ± 32.87	115.71 ± 35.62	131.33 ± 44.72
LDLC (Calculated)	97.65 ± 36.16	109.08 ± 41.64	84.77 ± 61.29
Mean difference	-0.25 ± 25.22	6.63 ± 26.43	46.55 ± 55.36
P-value	0.4580	<0.0001	<0.0001

Data presented as Mean±S.D P-value < 0.05 was considered statistically significant.

## DISCUSSION

The most important finding of our study on patients with diabetes mellitus is that the Friedewald equation tends to underestimate LDL-C. Friedewald formula is commonly used in most of routine clinical laboratories for the estimation of LDL-C. Various direct assays also developed to measure LDL-C.^2^,^11^,^12^ The determination of LDL-C is essential for the assessment of risk of cardiovascular disease and the treatment of dyslipidemia which is mostly based on strategies reducing LDL concentration, therefore LDL-C should be estimated accurately. The comparison of measured LDL-C and calculated LDL-C has shown different findings in different studies. Our study shows that calculated LDL-C by Friedewald formula underestimates the LDL level as compare to directly measured LDL-C at crucial points where accuracy is very essential. Same results were found in some other studies.^1^-^4^,^13^,^14^ Choi SY et al., found in their study that Friedewald formula extremely correlated with directly measured LDL-C, but the difference between two LDL-C values was approximately 11.51 mg/dl.^15^ Boshtam M et al., study demonstrated the Friedewald formula overestimated the LDL-C levels compared to the direct measurement method.^16^ According to Chai Kheng EY et al., in multiethnic Asian study population the negative bias of LDL-C is important especially when directly measured LDL-C is near the lower LDL-C cut-off used for risk categorization by using the Friedewald equation.^1^ Choi SY et al., suggest that alternative measurements of LDL-C could confuse clinicians especially in patients with diabetes mellitus.^15^ But according to Anwar M et al., many patients were classified in wrong NCEP cardiac risk categories by calculated methods of LDL-C determination.^2^

The negative bias of Friedewald-derived LDL-C was noted even at desirable triglyceride levels. Friedewald formula is very good at normal triglyceride levels and provides accurate results.^2^,^11^,^12^,^17^ The calculated methods did not have a uniform performance for LDL-C estimation at different triglyceride levels.^2^ Higher the triglyceride concentration, higher the negative bias in the Friedewald formula was observed.^13^ There was no statistically significant difference in calculated and measured LDL-C at triglyceride levels < 150 mg/dl, however, mean difference reached 6.63 and 46.55 at triglyceride levels between 150-400 mg/dl and >400 mg/dl respectively in our study. Same findings obtained from some other studies.^2^,^4^,^11^-^13^,^16^,^18^ This underestimation of LDL-C by calculated methods increased as triglyceride levels increased and many patients were classified in wrong cardiac risk categories. The direct assays are precise, accurate and not affected by triglyceride levels, therefore, should be used to measure LDL-C.^2^ The difference in direct and calculated LDL-C depends on serum triglyceride.^15^,^19^,^20^ The LDL derived from Friedewald equation is significantly underestimated at triglyceride concentrations >177 mg/dl.^13^,^16^,^18^ In another study, at triglyceride concentrations 177 mg/dl, the average LDL calculated by Friedewald equation was already 28% lower than direct LDL.^13^

## CONCLUSION

The findings of the study suggest that calculated LDL-C underestimates measured LDL-C, which may cause misclassifications that may have an impact on therapeutic decisions in patients with diabetes. Calculated LDL-C may depend on triglyceride levels hence LDL-C should be measured by direct assay in routine clinical laboratories.
